# Eliciting the Impact of Digital Consulting for Young People Living With Long-Term Conditions (LYNC Study): Cognitive Interviews to Assess the Face and Content Validity of Two Patient-Reported Outcome Measures

**DOI:** 10.2196/jmir.9786

**Published:** 2018-10-11

**Authors:** Jackie Sturt, Thandiwe Rebecca Dliwayo, Vera Forjaz, Kathryn Hamilton, Carol Bryce, Joseph Fraser, Frances Griffiths

**Affiliations:** 1 Florence Nightingale Faculty of Nursing, Midwifery and Palliative Care King's College London London United Kingdom; 2 Division of Psychiatry University College London London United Kingdom; 3 Research Department of Clinical, Educational and Health Psychology University College London London United Kingdom; 4 Warwick Medical School University of Warwick Coventry United Kingdom; 5 Joe's Diabetes Ltd London United Kingdom; 6 Centre for Health Policy University of the Witwatersrand Johannesburg South Africa

**Keywords:** communication, referral and consultation, electronic mail, text messaging, chronic disease, young adult, patient-reported outcome measures

## Abstract

**Background:**

Digital consulting, using email, text, and Skype, is increasingly offered to young people accessing specialist care for long-term conditions. No patient-reported outcome measures (PROMs) have been evaluated for assessing outcomes of digital consulting. Systematic and scoping reviews, alongside patient involvement, revealed 2 candidate PROMs for this purpose: the patient activation measure (PAM) and the physician’s humanistic behaviors questionnaire (PHBQ). PAM measures knowledge, beliefs, and skills that enable people to manage their long-term conditions. PHBQ assesses the presence of behaviors that are important to patients in their physician-patient interactions.

**Objective:**

This study aimed to assess the face and content validity of PAM and PHBQ to explore whether they elicit important outcomes of digital consulting and whether the PROMs can isolate the digital consultation component of care.

**Methods:**

Participants were drawn from 5 clinics providing specialist National Health Service care to 16- to 24-year-olds with long-term health conditions participating in the wider LYNC (Long-Term Conditions, Young People, Networked Communications) study. Overall, 14 people undertook a cognitive interview in this substudy. Of these, 7 participants were young people with either inflammatory bowel disease, cystic fibrosis, or cancer. The remaining 7 participants were clinicians who were convenience sampled. These included a clinical psychologist, 2 nurses, 3 consultant physicians, and a community youth worker practicing in cancer, diabetes, cystic fibrosis, and liver disease. Cognitive interviews were transcribed and analyzed, and a spreadsheet recorded the participants’ PROM item appraisals. Illustrative quotes were extracted verbatim from the interviews for all participants.

**Results:**

Young people found 11 of the PAM 13 items and 7 of the additional 8 PAM 22 items to be relevant to digital consulting. They were only able to provide spontaneous examples of digital consulting for 50% (11/22) of the items. Of the 7 clinicians, 4 appraised all PAM 13 items and 20 of the PAM 22 items to be relevant to evaluating digital consulting and articulated operationalization of the items with reference to their own digital consulting practice with greater ease than the young people. Appraising the PHBQ, in 14 of the 25 items, two-thirds of the young people’s appraisals offered digital consulting examples with ease, suggesting that young people can detect and discern humanistic clinician behaviors via digital as well as face-to-face communication channels. Moreover, 17 of the 25 items were appraised as relevant by the young people. This finding was mirrored in the clinician appraisals. Both young people and the clinicians found the research task complex. Young participants required considerably more researcher prompting to elicit examples related to digital consulting rather than their face-to-face care.

**Conclusions:**

PAM and PHBQ have satisfactory face and content validity for evaluating digital consulting to warrant proceeding to psychometric evaluation. Completion instructions require revision to differentiate between digital and face-to-face consultations.

## Introduction

### Clinical Outcomes in Young Adults

In the United Kingdom, 23% of 11- to 15-year-olds report having a long-term illness or disability and of those reporting a long-term condition, 58% take medication [1]. As these young people approach adulthood and take over greater responsibility for their health from their parents, their health outcomes notably decline. In type 1 diabetes, accessing care declines once they have transitioned from pediatric to adult services [2]. The key clinical outcome of importance to their future health, blood glucose control, deteriorates with 1 in 4 young people having blood glucose levels that will lead to complications of diabetes in adulthood [3,4]. In sickle cell disease, during adolescence, regular attendance at outpatient clinics reduces and poorer adherence to medical regimens, in particular penicillin prophylaxis, is reported [5-7]. This is especially problematic because 25% of young adult deaths due to sickle cell are linked to infection and poor compliance with penicillin prophylaxis [7]. In a birth to 18-year-old cohort with sickle cell, highest mortality occurred in those over 15 years with the transfer from pediatric to adult care settings. This represents a particularly high-risk situation [8]. In asthma, median rates of medication adherence in adolescents have been reported to be 43% [9], and when they present for emergency visits, they are less likely to continue treatment plans compared with younger children [10].

### Digital Consulting

In an attempt to improve poor health outcomes, specialist clinical teams caring for young people with long-term conditions have begun to use digital consulting, which commonly consists of email and text messaging [11]. This practice was investigated in the LYNC (Long-Term Conditions, Young People, Networked Communications) study that aimed to understand the role of digital consulting in specialist long-term condition clinics for 16- to 24-year-olds [12]. The LYNC study broadly defined digital consulting as two-way patient-clinician communications for clinical purposes, synchronous or asynchronous, in which either party is, or could be, mobile. The objective of this study was to explore how engagement in self-care is impacted by the use of digital consulting including effects, costs, and necessary safety and ethical safeguards. This was undertaken by interviewing and observing the practice of 173 clinical team members and 165 young people across 20 clinics providing care for young adults with 13 different physical and mental health conditions [11,13]. Young people and their clinicians value digital consulting as providing access to health care in a timely way and at a point when it makes a difference to how the young people manage their health [11].

### Patient-Reported Outcomes

Although these digital methods are increasingly being used by health professionals and are supported by health-provider organizations [14-16], their impact on patient-reported health outcomes has not been explored. In planning and undertaking health services improvement or experimental research, patient-reported outcomes (PROs) are of documented importance, and where you have a PRO, you need a patient-reported outcome measure (PROM) [17]. Examples of PROs are treatment satisfaction, depressive symptoms, quality of life, and pain.

Systematic reviews of digital consulting relevant to young people with long-term conditions identify gaps in our understanding of relevant PROs [12]. Digital consulting PROs need to demonstrate some amenability to change because of care being provided in both a face-to-face and digital contexts. For example, would the PRO be influenced if the young person had routine hospital appointments supplemented by email contact with one of their clinical teams between routine appointments? We might not expect depressive symptoms to change, but we might anticipate that treatment satisfaction could perhaps be influenced. Systematic reviews have also recommended undertaking research across several clinical populations using digital consulting [12]. To achieve this, a PRO that can span different long-term conditions and elicit generic outcomes related to the potential benefits of digital consulting is required.

### Identifying Candidate Patient-Reported Outcome Measures for Digital Consulting

As part of the LYNC study, a series of systematic and scoping reviews were undertaken alongside patient involvement to identify generic PROs and PROMs that assessed the impact of types of patient-clinician interactions (not specifically digital interactions) on patient engagement with their health as an outcome. This work is published elsewhere [13], but in summary, an initial systematic review to identify existing candidate generic PROMs to be used for evaluating digital consulting was undertaken. The review generated 28 generic PROMs from which we expected to identify an existing PROM that would be fit for purpose. To make the public and patient engagement task of appraising these PROMs manageable, they were clustered in PRO themes. The main PRO clusters were (1) quality of life (x 8 PROMs), (2) psychological status (x 5 PROMs), and (3) patient satisfaction with information technology (x 4 PROMs). Results of a Web-based public engagement survey (n=57) and discussion with the LYNC study public engagement team indicated that the PRO clusters were outdated and not fit for the purpose of evaluating the impact of digital consulting for young adults. Service user engagement in determining important outcomes of care is essential, and these discussions within the LYNC public engagement team were informed by the early LYNC fieldwork. This resulted in a decision to undertake a scoping review to identify interventions (and their PROMs) that aimed to improve face-to-face hospital appointments primarily by increasing patient participation in them. Two reviews were identified [18,19], and with reference to the emerging LYNC fieldwork data, the Harrington et al [18] review was particularly useful in informing a second Web-based public engagement survey to gain perspectives on the most important attributes of a successful face-to-face medical appointment. This survey (n=143) resulted in the emergence of 4 priorities. Using these as our guide, we returned to the Harrington et al review [18] to identify the PROMs that had been used to assess the impacts of interventions used in routine appointments to promote the following: (1) question asking, (2) engagement between the clinician and patient, (3) recall following an appointment, and (4) having a sense of disease control. None of the studies in the Harrington et al review [18] had utilized PROMs to assess these attributes specifically, but the scoping review, the public engagement process, and early LYNC fieldwork informed us about what we needed to look for in the PROM literature. A final scoping review identified 5 candidate PROMs that the LYNC public engagement team narrowed down to 3, which appeared potentially fit for the purpose of eliciting patient outcomes targeted by digital consulting and suitable for use across multiple long-term conditions. These PROMs were (1) the patient activation measure (PAM 22) [20], (2) the patient activation measure short form (PAM 13) [21], and (3) the physician’s humanistic behaviors questionnaire (PHBQ) [22]. The 13 items of the short form PAM 13 are contained within the 22 items of the PAM 22. Therefore, items from just 2 candidate PROMs, the PAM 22 and the PHBQ, were the focus of our interest.

### The Patient Activation Measure 22 and the Physician’s Humanistic Behaviors Questionnaire

The aim of PAM is to understand the knowledge, beliefs, and skills required by people to enable them to manage their long-term conditions. PAM 22 demonstrates construct and criterion validity [20]. PAM 13, a short-form version of PAM 22, has been more extensively examined for use in additional populations and languages [21]. It offers strong psychometric evidence in people living with long-term conditions [23-34]. PAM, specifically PAM-13, possesses a large body of evidence that supports its strong psychometric properties across diverse cultures and patient groups. PAM 13 and 22 are proprietorial PROMs that can limit access. The aim of PHBQ instrument development was to understand what physician humanistic behaviors, performed (or not) in the physician-patient interaction, were important to patients and to develop a measure to assess these behaviors in different health care contexts [22]. No further evaluations of the PHBQ full scale were identified, although a number of investigators have used items from PHBQ in new outcome measures or used PHBQ as a benchmark for convergent validity when developing new measures [35-37]. In summary, PHBQ evidences satisfactory face, content, and convergent validity but reports no reliability evidence. Research endeavor is required to address its psychometric properties relating to reliability and validity in other clinical contexts. A full assessment of the psychometric validation evidence for both PROMs is provided in Multimedia Appendix 1.

### Study Objectives

Neither PAM nor PHBQ have been used to evaluate the impact of digital consulting, and their face and content validity for this purpose have not been established. Our research objective was to assess the face and content validity of these measures for assessing the patient-clinician relationship impacts of digital consulting. In addition, where PROM items had face validity with young people and their clinicians, we also aimed to access whether they were thinking about the digital consultation component of their care when reflecting on their activation and their patient-clinician relationship.

## Methods

### Research Design

We undertook a qualitative exploration of PHBQ and PAM using cognitive interviewing, an established method for the development and revision of PROMs [38-42]. Criteria for PROM development and revision are that the items have clarity, relevance, and are unambiguous for their purpose [38]. Cognitive interviews use a concurrent interviewing approach where participants are asked to *think aloud* as they consider the measure or a debriefing approach where participants are asked to consider their experience of answering the questions and completing the measure immediately after its completion [38,39]. We used both concurrent and debriefing approaches in all interviews to understand the cognitions of the participants as they were considering individual items and their overall experience of each PROM and its fitness for purpose in the use of digital consulting. We acknowledged that there may be organizational barriers for clinicians in delivering care aligned with the individual PROM items in a digital care context. Consequently, it was important to expose each PROM to clinicians in addition to young people. This would identify whether there are differences in perspectives between people receiving digitally enhanced care and those offering it.

### Setting

A total of 5 clinics from the wider LYNC study participated in this substudy. LYNC study clinics spanned England and Wales and provided specialist National Health Service (NHS) care to 16- to 24-year-olds with long-term mental or physical health conditions [11,13]. The majority of clinicians and participants in the main LYNC study clinics were engaging in digital consultations in addition to routine clinic appointments.

### Participants

Interviewees were a subset of LYNC study participants with experience of digital consulting [12]. A total of 7 young people aged 16 to 24 years with a diagnosis of inflammatory bowel disease (4), cystic fibrosis (1), or cancer (2) consented to have a cognitive interview from a total sample of 165 young people recruited to the LYNC study. A total of 7 clinicians from 5 NHS trusts, also from the LYNC study pool of 173 clinical team members, participated. Of these, 1 was a clinical psychologist, 2 were nurses, 3 were consultant physicians, and 1 was a community youth worker. They worked in the fields of cancer (2), diabetes (2), cystic fibrosis (1), and liver disease (2). Convenience sampling was used in 5 of the recruited LYNC clinical sites. The complexity of the cognitive interviewing task meant that we needed young adult participants to agree to participate in a face-to-face interview. Everyone who was approached agreed to participate in the cognitive interview. We obtained ethical approval (14/WM/0066) from the National Research Ethics Service Committee West Midlands—The Black Country.

### Procedure

Three experienced researchers completed the interviews. Clinician participants were briefed on the purpose of the interview and the rationale for each candidate PROM. They were then asked to consider each item and articulate what they were considering as they read it. As each PROM was aimed at patient completion and not clinician completion, the clinicians were asked to consider the extent to which they themselves performed or supported the behaviors and attitudes in the items when thinking about digital consulting. They were asked to consider their own clinical care using the response options included in each measure (eg, strongly agree to strongly disagree). In doing so, they would be prompted to consider the item’s relevance in a digital consulting context. A total of 5 clinicians reviewed both PROMs and 2 reviewed 1 PROM each. Young people’s PROM cognitive interviews took place at the end of a shortened regular LYNC interview [11]. The young participants were briefed on the purpose of the interview and of the measure. To reduce participant burden, each young person was asked to consider, in rotation, 1 in 3 of the items in both measures. The sequential rotation of shortened questionnaires was documented to ensure item coverage during interviews. Young people were primed when considering the measures to think about their current access to, and communication with, their health care team, including digital consultations, and how completely they felt the questions addressed the sorts of things that are important to them in terms of engaging in digital consultations. We expected that young people would find it difficult to separate out their face-to-face interactions from their digitally enhanced care. Consequently, young people were asked to *think aloud* as they considered the items in the measure in relation to their own care and articulate a personal response to each item, and where necessary, they were prompted to clarify whether they were appraising in terms of their experience of digital consultations. Both clinicians and patients were also prompted to reflect on the measures in their entirety after having reviewed the individual items in terms of the overall relevance of the items for assessing the outcomes of digital consultations and whether there were any important aspects of this assessment that were currently omitted. All interviews were audio recorded, transcribed verbatim, and anonymized.

### Analysis

For both young person and clinician interviews, a spreadsheet was developed to indicate, by item, whether the participant could appraise the item as related to digital consulting with ease, operationalized by offering examples as they *thought aloud*, and whether the item was considered relevant. Illustrative quotes were extracted verbatim from the transcribed interviews for all participants. Where 2 or more young people and 4 or more clinicians appraised the item as relevant, we determined the item to be relevant.

## Results

### Face and Content Validity Assessments of the Patient Activation Measure 13 and 22 Items

PAM 22 was reviewed in entirety by 6 of the 7 clinicians, and selected items were reviewed in rotation by 7 young people. Each individual item of PAM 22 was reviewed a minimum of 8 times overall. Multimedia Appendix 2 presents the degree to which young peoples’ and clinicians’ responses indicated that they agreed or strongly agreed with the relevance of each item as an outcome of digital consulting. It also presents the degree to which digitally enhanced care was referred to spontaneously with ease or when prompted while considering the relevance of the item operationalized as examples being given in the interviews. Illustrative quotes from the cognitive interviews show where digital consulting was shown to be underpinning the construct of patient activation.

#### Young People

Multimedia Appendix 2 illustrates that in comparison with clinicians, the young people were less able to appraise the items as related to digital consulting with ease. In only 50% (11/22) of the items was more than 1 young person, out of the 2 or 3 reviewing the items, able to give an illustrative *think aloud* example. This did not differ according to whether they were appraising PAM 13 or PAM 22 items. Young people had less difficulty with appraising item relevance for digital consulting. On PAM 13, 11 of the items were assessed as being relevant to young peoples’ experience of managing their health condition with digital access to clinicians. Of the additional 8 PAM 22 items, 7 were found to be relevant. In articulating digital examples, the young people provided a similar proportion of these across all of the PAM 13 and PAM 22 items identified to be relevant. We had anticipated that young people would struggle to critique the measure items and instead use the item as a mechanism for appraising their abilities or their care. In appraising both the PAM 13 and 22 items, we found that young people did default to this position while undertaking this complex task. For example, in the last quote in Multimedia Appendix 2, the young person offers evidence of how they demonstrate that they can handle symptoms at home rather than on whether being able to handle their symptoms at home may be facilitated by the use of digital consulting. One young person reported that some of the questions were similar to others. Another suggested that PAM 22 could be used to assess and compare whether young people preferred digital versus face-to-face communication. The complex nature of the task limited the extent to which the young people were able to critique the PAM scale overall in depth and comment on item duplication or item omissions in important areas related to digital consulting.

#### Clinicians

Clinician participants had an easier grasp of the task than the young people and were able to appraise the items with relative ease. At least 4 of the 6 clinicians were able to address the items as related to digital consulting with ease on all PAM 13 items and 20 of the PAM 22 items. The majority of clinicians agreed that the measure was suitable for appraising digital consulting. For example, on PAM 13, if all 6 clinicians had agreed with the relevance of all 13 items, a 100% agreement rate would have been established by a total agreement score of 78. The agreement score was 63, which indicates high levels of agreement across the items and the clinicians on the item relevance for evaluating digital consulting. Most were able to provide examples of the relevance of the items to digitally enhanced care in support of their assessment. The clinicians were more able to critique the PAM items beyond the direct task and raised a number of issues. In relation to some items on the PAM 22 scale, with item similarities observed in the following paired questions—item 1 and 2; 13 and 16; and 18 and 20—made either item redundant. A diabetes consultant commented that they struggled to establish the relevance of item 3 and 6 in relation to evaluating outcomes for digital consulting and suggested that further clarification was required. The same clinician also remarked about the significant overlap between item 10, 12, and other items in the instrument. A liver clinician also found items 15 and 20 to be ambiguous and therefore could not provide a response.

Two clinicians suggested that there were items that may be more or less applicable for particular conditions. For example, a clinician suggested that item 18 on PAM 22 was condition-specific and would yield different responses in various health conditions. The young people’s community worker in cancer services disagreed with the idea in item 1 inferring that the patient should take sole responsibility for their health condition and reported that it took a joint effort to maintain good health outcomes for each patient. The diabetes consultant reported that they were yet to encounter a patient who had successfully made changes in adopting a healthy lifestyle when considering item 16 on the PAM 22 scale. A young people’s community worker in cancer services commented that the measure appeared sufficiently comprehensive by covering a broad context of how technology might contribute to supporting self-management and found items 4 and 8 to be particularly relevant. However, this health professional added that not all items of PAM 22 would be relevant to all patient groups. This idea was supported by a cystic fibrosis consultant who reported that although they strongly agreed with the first 6 items on the scale, some items relating to taking sole action of one’s health condition would be less important for a cystic fibrosis patient group than for other health conditions. The clinician reported that due to the nature of the health condition, young people living with cystic fibrosis were usually knowledgeable about managing their health condition at home and infrequently visited their general physicians in the community.

A lead cancer nurse summarized that PAM 22 adequately probed the right issues in terms of assessing the outcomes of digital consulting. This idea was also supported by a diabetes consultant, but he/she concluded that although the instrument was exhaustive, it was quite repetitive. Some concepts were missing for some clinicians in confirming the PAM 13 or 22 as a comprehensive measure of the effects of digital consulting. The liver clinician stated that it was paramount for any assessment to include items that would gauge whether there were notable changes in young people’s confidence attributed to digital consulting. Overall, PAM was appraised as having a satisfactory level of face and content validity notwithstanding the issues raised regarding some of the items.

### Face and Content Validity Assessments of the Physician Humanistic Behaviors Questionnaire

Assessments were made by 6 of the 7 clinicians and 7 young people, and each individual item was assessed a minimum of 8 times. Multimedia Appendix 3 presents data of item relevance and illustrative examples where digital consulting was identified to be underpinning the constructs and/or outcomes of physician humanistic behaviors.

#### Young People

Compared with the clinicians, the 7 young people appraising these items found it very difficult to engage in a lengthy critique. Young people had a tendency to answer the question rather than articulate its relevance to digital consulting. The young people required prompting to clarify whether their responses were underpinned by digital consulting, and on some occasions, they could not elaborate any further beyond one-word responses when prompted about this. Nonetheless, Multimedia Appendix 3 illustrates that for 56% (13/25) of the items, at least 2 of the 3 young people reviewing each item were able to offer digital examples with ease. This suggests that young people can detect and discern humanistic clinician behaviors via digital as well as face-to-face communication channels. In terms of item relevance, 68% (17/25) of the items were apprised as relevant by 2 or more young people. As with the PAM appraisal, the complexity of the task limited a wider critique on item repetition or omission by the young people.

#### Clinicians

If all 6 clinician appraisals had agreed with the relevance of all 25 items, a 100% agreement rate would have been established, resulting in a total agreement score of 150. The agreement score was 108, which indicates reasonable levels of agreement across the items and the clinicians on the item relevance for evaluating digital consulting. Clinicians were able to provide examples of how the items elicited aspects of their performance relating to digital consulting. There were queries raised with regard to the ambiguity and applicability of some items on PHBQ. They were concerned that the idea of making promises to patients in item 7 was uncomfortable and suggested that the item needed readjustment. A diabetes clinician reported that item 3, *is in a hurry*, required a context to be given to make the question clear for the patients to respond. Moreover, 3 clinicians had queries regarding the relevance of item 21. The professionals stated that they were already familiar with their patients and would know how they preferred to be addressed. One clinician agreed with the relevance of item 1, which probed clinicians’ ability to follow through on patients’ problems and provided examples of how this aspect of performance related to digital consulting. Item 11 was thought to be irrelevant with regard to evaluating digital consulting by the same health professional, and this idea was also supported by a cystic fibrosis consultant who expressed a similar opinion with regard to item 16. These 2 professionals considered item 17 to be inappropriate for a digital consulting scenario, although 1 clinician appreciated that abruptness could be communicated in an email.

A small number of recommendations were made to either add or alter existing items on the scale. Two clinicians suggested that the addition of items that probed whether clinicians respond to queries in a timely manner would be valuable. An individual expressed concern that PHBQ appeared to scrutinize clinicians’ behaviors without much consideration of young people's conduct toward health professionals. They suggested that the instrument needed to account for the prior agreement established between health professionals and young people with respect to methods of communication and essentially probe those dynamics. They concluded that PHBQ was useful in asking the right questions to obtain young people’s opinions about the outcome of digital consulting in terms of the clinician’s behavior.

## Discussion

### Principal Findings

Cognitive interviews with 7 clinicians and 7 young people found that PAM and PHBQ contain items that were satisfactorily appraised by clinicians and patients as having reasonable degrees of face and content validity. In addition, both scales have demonstrated themselves to be credible clinical evaluation measures for assessing the effects of digital consulting. The young people found it difficult to separate their digital experiences from their face-to-face consultation experiences. The clinicians had less difficulty with this task and context. Each young person’s experience is limited by the n-of-1 nature of their clinical experiences, whereas the clinicians have multiple young patients upon whom they were drawing to produce illustrative examples with ease.

### Strengths and Limitations of the Research Methods

The cognitive interviews provided in-depth analyses of 2 PROMs with both clinicians and patients. We recruited a broad range of health professional disciplines to participate, and they were experienced providers of digitally enhanced care. Consequently, they were evaluating the measures’ real clinical relevance for use in a service evaluation or audit context. In relation to the young people, they were all receivers of digitally enhanced care, and all patients evaluated some aspects of both measures. These participants were conveniently sampled, and their representativeness of the whole LYNC study population cannot be assured. Despite 4 of the main LYNC case study sites being mental health services, none of the cognitive interviews were undertaken with clinicians or young people from a mental health service, and this is a limitation of the evidence presented. The most apparent methodological weakness is that most young people and some clinicians did not understand the exercise of having a cognitive interview and the additional challenge of distinguishing their digital and face-to-face consulting experiences. The interviewers had to work hard with the time and level of understanding that each participant presented. In this respect, it could be argued that some interviews were not sufficiently accessing young people's, autonomous cognitions on the topic. Often, considerable prompting from the researcher was required to facilitate the young person’s understanding of the interview purpose and elicit the required data. The PHBQ scale was developed for the purpose of appraising physician behaviors, and we applied it to a broader range of health professionals. Despite this original focus, no participant referred to any limitation of this broader application.

### Strengths and Limitations of the Patient-Reported Outcome Measures

Previous research has demonstrated that psychometrically, PAM 13 has stronger and more detailed all-round validity in demonstrating clear face, content, construct, and criterion validity and psychometric reliability across populations [21,23-34]. A large body of evidence, therefore, demonstrates that PAM-13 is a valid, reliable, and clinically useful measure of patient activation that can be used across diverse cultures and patient groups. In light of the evidence presented above, the strong psychometric properties of PAM 13, in particular, support its use in adults with long-term conditions. Its validity in a digital context has not previously been established in the literature. PAM 22 has been psychometrically appraised fewer times [20]. The PAM 22 and 13 outcomes in young adult populations have been reported [43-45], although Disabato et al [45] questioned the appropriateness of PAM 13 in this population. Its psychometric evaluation in a young adult German population with a mean age of 17.5 years has however since been satisfactorily established [46]. The PHBQ has less documented detail about the face and content validity for face-to-face consultations in a single study appraising its psychometric properties [22]. A revised version of PHBQ was used in a study of 1400 service men and women in which 41% of the study population were aged 18 to 24 years [47]. The authors reported no methodological limitation in the use of the measure with their participants.

During interviews, clinician participants were not specifically directed to appraise both PROMs as research tools and in their principal roles as clinicians; they may have adopted a view of them as clinical tools. This may have contributed to some negative appraisals regarding item or topic repetition within the scale. Asking the same question in different ways is often a strength in a research tool to enable the research team to tease out response congruity. In a clinical setting, where time for both clinicians and patients is at a premium, repetition may appear wasteful and irritating. Our LYNC findings point to the role of digital consulting as having particular strength when there is a pre-existing relationship between the patient and the clinician [11]. This aligns well with the PHBQ scale, which is assessing physician humanistic behaviors, where 2 previous face-to-face consultations are required before scale completion.

### Comparisons With Prior Work

PAM is one of the few PROMs that serves twin purposes of performing well as both a clinical and research tool. Armstrong et al (2017) evaluated the use of PAM as a clinical tool in patient care and found it to prompt a range of both positive and negative responses by both patients and clinicians and concluded that further evaluative work in its clinical application is warranted [48]. PROMs are increasingly important across the health care sector. The US Food and Drug Administration (FDA) [49] has produced guidance on their use by the pharmaceutical industry in recognition that the evolution of science and health care products and practices should not come at any price. There is concern that drugs can have detrimental effects on the constructs measured by PROMs, and it is important to identify this early in the evaluation of the drug so that it can be optimized to reduce patient treatment burden, for example, on quality of life. Although this advice is specifically intended for the pharmaceutical industry, Speight and Barendse [17] remind us that the principles underpinning the FDA’s guidance apply across other contexts. Health care is awash with technological solutions, and in the area of digital consulting, NHSmail2, a secure email service for sharing patient identifiable and sensitive information, is currently undergoing a rollout across the NHS with the specific objective of promoting digital communication between many users, including patients. With such fundamental shifts in the way that health care will be delivered and received on the horizon, it is imperative that we ensure we have clinically and scientifically fit-for-purpose PROMs available that have credibility with patients, clinicians, researchers, and technological innovators.

### Recommendations for Research

The strength of PAM 13 evidence indicates that it represents the best-evidenced PROM for evaluating the impact of digital consulting on patient activation behaviors. Our research indicates that revision of the completion instructions would be necessary to enable participants or patients to differentiate between digital and face-to-face consultation in their responses. Subsequent research studies need to expose PAM 13, 22, and PHBQ to clinical evaluations of digitally enhanced services to examine how they perform psychometrically in a larger population. Face and content validity of these PROMs need to be explored in mental health settings delivering digitally enhanced care. Although we acknowledge that these PROMs may not be perfect and may benefit from item addition, revision, and removal, they are presented as the best PROMs available at the current time to evaluate the impact of digital consulting for young adults with long-term conditions. The development of a new PROM for this purpose may be appropriate in the future.

## Appendix

Multimedia Appendix 1 Strength of validity and reliability evidence for the candidate patient-reported outcome measures.

Multimedia Appendix 2 Clinician and young people’s face and content validity assessments of the Patient Activation Measure 13 and 22 items.

Multimedia Appendix 3 Clinicians and young people’s face and content validity assessments of the Physician Humanistic Behavior Questionnaire.

## Abbreviations

FDA: Food and Drug Administration
NHS: National Health Service
PAM: patient activation measure
PHBQ: physician’s humanistic behaviors questionnaire
PRO: patient-reported outcome
PROM: patient-reported outcome measure

## References

[1] Brooks F, Magnusson J, Klemera E, Chester K, Spencer N, Smeeton N (2015). HBSC England National Report: Health Behaviour in School-aged Children HBSC: World Health Organization Collaborative Cross National Study.

[2] (2016). Royal College of Paediatric and Child Health.

[3] (2012). Royal College of Paediatrics and Child Health.

[4] (2016). Digibete.

[5] Witherspoon D, Drotar D (2010). Correlates of adherence to prophylactic penicillin therapy in children with sickle cell disease. Child Health Care.

[6] Howard J, Woodhead T, Musumadi L, Martell A, Inusa BP (2010). Moving young people with sickle cell disease from paediatric to adult services. Br J Hosp Med (Lond).

[7] Musumadi L, Westerdale N, Appleby H (2012). An overview of the effects of sickle cell disease in adolescents. Nurs Stand.

[8] Quinn CT, Rogers ZR, McCavit TL, Buchanan GR (2010). Improved survival of children and adolescents with sickle cell disease. Blood.

[9] Naimi DR, Freedman TG, Ginsburg KR, Bogen D, Rand CS, Apter AJ (2009). Adolescents and asthma: why bother with our meds?. J Allergy Clin Immunol.

[10] Cooper WO, Hickson GB (2001). Corticosteroid prescription filling for children covered by Medicaid following an emergency department visit or a hospitalization for asthma. Arch Pediatr Adolesc Med.

[11] Griffiths F, Bryce C, Cave J, Dritsaki M, Fraser J, Hamilton K, Huxley C, Ignatowicz A, Kim SW, Kimani PK, Madan J, Slowther AM, Sujan M, Sturt J (2017). Timely digital patient-clinician communication in specialist clinical services for young people: a mixed-methods study (The LYNC study). J Med Internet Res.

[12] Griffiths FE, Atherton H, Barker JR, Cave JA, Dennick K, Dowdall P, Fraser J, Huxley C, Kim SW, Madan JJ, Matharu H, Musumadi L, Palmer TM, Paul M, Sankaranarayanan S, Slowther AM, Sujan MA, Sutcliffe PA, Sturt J (2015). Improving health outcomes for young people with long term conditions: the role of digital communication in current and future patient-clinician communication for NHS providers of specialist clinical services for young people - LYNC study protocol. Digit Health.

[13] Griffiths FE, Armoiry X, Atherton H, Bryce C, Buckle A, Cave JA, Court R, Hamilton K, Dliwayo TR, Dritsaki M, Elder P, Forjaz V, Fraser J, Goodwin R, Huxley C, Ignatowicz A, Karasouli E, Kim SW, Kimani P, Madan JJ, Matharu H, May M, Musumadi L, Paul M, Raut G, Sankaranarayanan S, Slowther AM, Sujan MA, Sutcliffe PA, Svahnstrom I, Taggart F, Uddin A, Verran A, Walker L, Sturt J (2018). The role of digital communication in patient-clinician communication for NHS providers of specialist clinical services for young people The LYNC study: a mixed methods study. NIHR Journals Library.

[14] (2012). Department of Health.

[15] Wachter R (2016). The Wachter Review of Health IT: Update and Insights. The King's Fund.

[16] Wachter RM (2016). Making IT work: harnessing the power of health information technology to improve care in England.

[17] Speight J, Barendse SM (2010). FDA guidance on patient reported outcomes. BMJ.

[18] Harrington J, Noble LM, Newman SP (2004). Improving patients' communication with doctors: a systematic review of intervention studies. Patient Educ Couns.

[19] Pendleton D, Hasler J (1983). Doctor-Patient Communication.

[20] Hibbard JH, Stockard J, Mahoney ER, Tusler M (2004). Development of the Patient Activation Measure (PAM): conceptualizing and measuring activation in patients and consumers. Health Serv Res.

[21] Hibbard JH, Mahoney ER, Stockard J, Tusler M (2005). Development and testing of a short form of the patient activation measure. Health Serv Res.

[22] Weaver MJ, Ow CL, Walker DJ, Degenhardt EF (1993). A questionnaire for patients’ evaluations of their physicians’ humanistic behaviors. J Gen Intern Med.

[23] Rademakers J, Nijman J, van der Hoek L, Heijmans M, Rijken M (2012). Measuring patient activation in The Netherlands: translation and validation of the American short form Patient Activation Measure (PAM13). BMC Public Health.

[24] Skolasky RL, Green AF, Scharfstein D, Boult C, Reider L, Wegener ST (2011). Psychometric properties of the patient activation measure among multimorbid older adults. Health Serv Res.

[25] Zill JM, Dwinger S, Kriston L, Rohenkohl A, Härter M, Dirmaier J (2013). Psychometric evaluation of the German version of the Patient Activation Measure (PAM13). BMC Public Health.

[26] Schmaderer M, Pozehl B, Hertzog M, Zimmerman L (2015). Psychometric properties of the Patient Activation Measure in multimorbid hospitalized patients. J Nurs Meas.

[27] Green CA, Perrin NA, Polen MR, Leo MC, Hibbard JH, Tusler M (2010). Development of the Patient Activation Measure for Mental Health. Adm Policy Ment Health.

[28] Moljord IE, Lara-Cabrera ML, Perestelo-Pérez L, Rivero-Santana A, Eriksen L, Linaker OM (2015). Psychometric properties of the Patient Activation Measure-13 among out-patients waiting for mental health treatment: A validation study in Norway. Patient Educ Couns.

[29] Maindal HT, Sokolowski I, Vedsted P (2009). Translation, adaptation and validation of the American short form Patient Activation Measure (PAM13) in a Danish version. BMC Public Health.

[30] Ahn YH, Yi CH, Ham OK, Kim BJ (2015). Psychometric properties of the Korean version of the Patient Activation Measure 13 (PAM13-K) in patients with osteoarthritis. Eval Health Prof.

[31] Stepleman L, Rutter MC, Hibbard J, Johns L, Wright D, Hughes M (2010). Validation of the patient activation measure in a multiple sclerosis clinic sample and implications for care. Disabil Rehabil.

[32] Packer TL, Kephart G, Ghahari S, Audulv Å, Versnel J, Warner G (2015). The Patient Activation Measure: a validation study in a neurological population. Qual Life Res.

[33] Hung M, Carter M, Hayden C, Dzierzon R, Morales J, Snow L, Butler J, Bateman K, Samore M (2013). Psychometric assessment of the patient activation measure short form (PAM-13) in rural settings. Qual Life Res.

[34] Brenk-Franz K, Hibbard J, Herrmann W, Freund T, Szecsenyi J, Djalali S, Steurer-Stey C, S&ouml;nnichsen A, Tiesler F, Storch M, Schneider N, Gensichen J (2013). Validation of the German version of the patient activation measure 13 (PAM13-D) in an international multicentre study of primary care patients. PLoS One.

[35] Glaser KM, Markham FW, Adler HM, McManus PR, Hojat M (2007). Relationships between scores on the Jefferson Scale of physician empathy, patient perceptions of physician empathy, and humanistic approaches to patient care: a validity study. Med Sci Monit.

[36] Dine CJ, Ruffolo S, Lapin J, Shea JA, Kogan JR (2014). Feasibility and validation of real-time patient evaluations of internal medicine interns' communication and professionalism skills. J Grad Med Educ.

[37] She M, Li Z, Rau PL (2015). Physician Communication Behaviors that Predict Patient Trust in Outpatient Departments.

[38] Ahmed N, Bestall JC, Payne SA, Noble B, Ahmedzai SH (2009). The use of cognitive interviewing methodology in the design and testing of a screening tool for supportive and palliative care needs. Support Care Cancer.

[39] Drennan J (2003). Cognitive interviewing: verbal data in the design and pretesting of questionnaires. J Adv Nurs.

[40] Rosal MC, Carbone ET, Goins KV (2003). Use of cognitive interviewing to adapt measurement instruments for low-literate Hispanics. Diabetes Educ.

[41] Knafl K, Deatrick J, Gallo A, Holcombe G, Bakitas M, Dixon J, Grey M (2007). The analysis and interpretation of cognitive interviews for instrument development. Res Nurs Health.

[42] Cooke D, O'Hara MC, Beinart N, Heller S, La Marca R, Byrne M, Mansell P, Dinneen SF, Clark M, Bond R, Speight J, UK NIHR DAFNE Study Group (2013). Linguistic and psychometric validation of the Diabetes-Specific Quality-of-Life Scale in UK English for adults with type 1 diabetes. Diabetes Care.

[43] Flink M, Lindblad M, Frykholm O, Kneck Å, Nilsen P, Årestedt K, Ekstedt M (2017). The Supporting Patient Activation in Transition to Home (sPATH) intervention: a study protocol of a randomised controlled trial using motivational interviewing to decrease re-hospitalisation for patients with COPD or heart failure. BMJ Open.

[44] Huang JS, Terrones L, Tompane T, Dillon L, Pian M, Gottschalk M, Norman GJ, Bartholomew LK (2014). Preparing adolescents with chronic disease for transition to adult care: a technology program. Pediatrics.

[45] Disabato JA, Cook PF, Hutton L, Dinkel T, Levisohn PM (2015). Transition from pediatric to adult specialty care for adolescents and young adults with refractory epilepsy: a quality improvement approach. J Pediatr Nurs.

[46] Bomba F, Markwart H, Mühlan H, Menrath I, Ernst G, Thyen U, Schmidt S (2018). Adaptation and validation of the German Patient Activation Measure for adolescents with chronic conditions in transitional care: PAM 13 for Adolescents. Res Nurs Health.

[47] Douglas SR, Vides de Andrade AR, Boyd S, Leslie M, Webb L, Davis L, Fraine M, Frazer NL, Hargraves R, Bickman L (2016). Communication training improves patient-centered provider behavior and screening for soldiers' mental health concerns. Patient Educ Couns.

[48] Armstrong N, Tarrant C, Martin G, Manktelow B, Brewster L, Chew S (2017). Independent evaluation of the feasibility of using the Patient Activation Measure in the NHS in England-Final report.

[49] Patrick DL, Burke LB, Powers JH, Scott JA, Rock EP, Dawisha S, O'Neill R, Kennedy DL (2007). Patient-reported outcomes to support medical product labeling claims: FDA perspective. Value Health.

