# Supplementary material for: Eliciting the Impact of Digital Consulting for Young People Living With Long-Term Conditions (LYNC Study): Cognitive Interviews to Assess the Face and Content Validity of Two Patient-Reported Outcome Measures
Source: J Med Internet Res. 2018 Oct 11;20(10):e268. doi: 10.2196/jmir.9786 (PMC6231804; doi:10.2196/jmir.9786)
Supplement: Multimedia Appendix 1 [file jmir_v20i10e268_app1.pdf]

Searches were undertaken of PubMed, EMBASE, PsychInfo, PsychArticles and Medline using each measure title as search terms i.e. Patient Activation Measure and Physicians' Humanistic Behavioural Questionnaire to identify scale validation publications. This evidence is summarised below and helps to contextualise and support the findings and conclusions of the empirical evidence for their use in digital consulting.

The Patient Activation Measure (PAM)

The aim of the PAM instrument development was to understand the knowledge, beliefs and skills required by people to enable them to manage their long term condition. PAM development commenced with a literature review from which a Delphi type expert consensus process was completed with 21 experts (1). Six domains were subsequently developed and presented to 19 service users in two focus groups for further exploration. Using the 6 domains a pool of 80 items was generated and these provided the basis for three rounds of cognitive interviews with 20 participants. A 75 item measure resulted that was then completed by 100 participants. Rasch analysis was undertaken on the data which resulted in a 21-item measure with a reliability of 0.85 (real) and 0.87 (model) and Cronbach's alpha of 0.87. A test-retest reliability assessment conducted by 30 respondents two weeks after the initial interview found that 28 out of 30 respondents had a retest activation estimate within 95% CI of their test activation estimate. The third stage of extending and refining the PAM achieved a Rasch person reliability of 0.85 (real), 0.88 (model) and a Cronbach's alpha of 0.91. When tested with a national sample at stage four, the Rasch person reliability was 0.87 (real) and 0.91 (model) (1). Criterion validity was examined with 10 respondents from the initial study selected based on recording lowest and highest responses. In-depth semi-structured interviews were conducted with these participants with the aim of obtaining elaborate responses, and three judges who were blinded to the participant's measured activation, independently reviewed and categorised each transcript as either 'low' or 'high.' Results illustrated that the three independent judges' classification of participants agreed with the measured activation level 83% of the time. Cohen's kappa for measured activation and each judges' classification were 0.80, 0.90 and 0.90. In a third stage new items were added to challenge respondents at the top and bottom ends on the activation scale resulting in a 22-item scale and this was evaluated in a further 486 participants with and without a long term condition. In a final stage, the PAM was delivered in a national probability sample of 1,515 people over 45 yrs. The PAM demonstrated construct and criterion validity as individuals with higher activation reported significantly better health as measured by the SF-8, lower rates of doctor appointments, emergency room visits and hospital nights ($r = -0.07$, $p < 0.01$). Those with higher activation were also more likely to engage in healthy behaviours (1).

The PAM 13, a short-form version of the PAM 22, has been more extensively examined for use in additional populations and languages (2). It offers strong psychometric evidence in populations relevant to the LYNC study population as follows; adults with long term physical conditions (3), elderly adults with multi-morbidity (4, 5), in adults with multi-morbidity (6), long term mental health conditions (7, 8), diabetes and dysglycemia (9), osteoarthritis (10), neurological conditions (11, 12), patients with chronic disease living in rural areas (13) and primary care settings (14). A large body of evidence therefore demonstrates that the PAM-13 is a valid, reliable and clinically useful measure of patient activation that can be used across diverse cultures and patient groups. In light of the evidence presented above; the strong psychometric properties of the PAM 13 in particular support its use in adults with long term conditions. Both the PAM 13 and 22 are proprietorial PROMS which can limit access to these PROMS.

Physician Humanistic Behaviour Scale (PHBQ)

The aim of the PHBQ instrument development was to understand what humanistic physician behaviours, performed (or not) in the physician-patient interaction, were important to patients and to develop a measure to assess for these behaviours in different health care contexts. (15) Participating patients were required to have previously consulted with a named physician on at least two previous occasions. During a process of structured interviews and questionnaires patients were asked to define and give examples of behaviours that communicated, or not, integrity, respect and compassion. From these interviews, the authors developed 96 items. Through a further process of face and content validity assessment with 96 patients, and completion by further 109 patients, the 25 item PHBQ was confirmed and administered along with the Medical Interview Satisfaction Scale (16) to 119 medical in-patients and 111 medical outpatients to assess the humanistic behaviours of 6 junior physicians. A senior physician rated the humanistic behaviours of the junior physicians once during the study period. The study demonstrates convergent reliability in the close correlation between the PHBQ and the Medical Interview Satisfaction Scale in both settings ($r=0.87$, $p < 0.001$) indicating that humanistic physician behaviour increases satisfaction with care evaluations by patients. This conclusion is supported by a systematic review which includes the PHBQ (17). Humanistic assessments of physicians were higher in the out-patient clinic setting than the in-patient hospital setting. The authors indicate a number of possible reasons for this; the physicians behave differently in the two settings, possibly due to organisational and workload pressures, and the patients have different expectations of

their physicians in each setting. No further evaluations of the PHBQ full scale were found in the literature although a number of investigators have used items from the PHBQ in new outcome measures or used the PHBQ as a bench mark for convergent validity when developing new measures (18-20). In conclusion, the PHBQ evidences satisfactory face, content and convergent validity but reports no reliability evidence. Research endeavour is required to address its psychometric properties relating to reliability and validity in other clinical contexts.

References

1. Hibbard JH, Stockard J, Mahoney ER, Tusler M. Development of the Patient Activation Measure (PAM): conceptualizing and measuring activation in patients and consumers. Health services research. 2004;39(4p1):1005-26.
2. Hibbard JH, Mahoney ER, Stockard J, Tusler M. Development and testing of a short form of the patient activation measure. Health services research. 2005;40(6p1):1918-30.
3. Rademakers J, Nijman J, van der Hoek L, Heijmans M, Rijken M. Measuring patient activation in the Netherlands: translation and validation of the American short form Patient Activation Measure (PAM13). BMC Public Health. 2012;12(1):1.
4. Skolasky RL, Green AF, Scharfstein D, Boult C, Reider L, Wegener ST. Psychometric properties of the patient activation measure among multimorbid older adults. Health services research. 2011;46(2):457-78.
5. Zill JM, Dwinger S, Kriston L, Rohenkohl A, Härter M, Dirmaier J. Psychometric evaluation of the German version of the patient activation measure (PAM13). BMC public health. 2013;13(1):1.
6. Schmaderer M, Pozehl B, Hertzog M, Zimmerman L. Psychometric Properties of the Patient Activation Measure in Multimorbid Hospitalized Patients. Journal of Nursing Measurement. 2015;23(3):128E-41E.
7. Green CA, Perrin NA, Polen MR, Leo MC, Hibbard JH, Tusler M. Development of the Patient Activation Measure for mental health. Administration and Policy in Mental Health and Mental Health Services Research. 2010;37(4):327-33.
8. Moljord IEO, Lara-Cabrera ML, Perestelo-Pérez L, Rivero-Santana A, Eriksen L, Linaker OM. Psychometric properties of the Patient Activation Measure-13 among out-patients waiting for mental health treatment: A validation study in Norway. Patient education and counseling. 2015;98(11):1410-7.
9. Maindal HT, Sokolowski I, Vedsted P. Translation, adaptation and validation of the American short form Patient Activation Measure (PAM13) in a Danish version. BMC Public Health. 2009;9(1):1.
10. Ahn Y-H, Yi C-H, Ham O-K, Kim B-J. Psychometric properties of the Korean version of the “Patient Activation Measure 13”(PAM13-K) in patients with osteoarthritis. Evaluation & the health professions. 2015;38(2):255-64.
11. Stepleman L, Rutter M-C, Hibbard J, Johns L, Wright D, Hughes M. Validation of the patient activation measure in a multiple sclerosis clinic sample and implications for care. Disability and rehabilitation. 2010;32(19):1558-67.
12. Packer TL, Kephart G, Ghahari S, Audulv Å, Versnel J, Warner G. The Patient Activation Measure: a validation study in a neurological population. Quality of Life Research. 2015;24(7):1587-96.

13. Hung M, Carter M, Hayden C, Dzierzon R, Morales J, Snow L, et al. Psychometric assessment of the patient activation measure short form (PAM-13) in rural settings. Quality of Life Research. 2013;22(3):521-9.
14. Brenk-Franz K, Hibbard JH, Herrmann WJ, Freund T, Szecsenyi J, Djalali S, et al. Validation of the German version of the patient activation measure 13 (PAM13-D) in an international multicentre study of primary care patients. PloS one. 2013;8(9):e74786.
15. Weaver MJ, Ow CL, Walker DJ, Degenhardt EF. A questionnaire for patients' evaluations of their physicians' humanistic behaviors. Journal of General Internal Medicine. 1993;8(3):135-9.
16. Wolf MH, Putnam SM, James SA, Stiles WB. The medical interview satisfaction scale: development of a scale to measure patient perceptions of physician behavior. Journal of behavioral medicine. 1978;1(4):391-401.
17. Boon H, Stewart M. Patient-physician communication assessment instruments:: 1986 to 1996 in review. Patient education and counseling. 1998;35(3):161-76.
18. Glaser KM, Markham FW, Adler HM, McManus PR, Hojat M. Relationships between scores on the Jefferson Scale of physician empathy, patient perceptions of physician empathy, and humanistic approaches to patient care: a validity study. Medical Science Monitor. 2007;13(7):CR291-CR4.
19. Dine CJ, Ruffolo S, Lapin J, Shea JA, Kogan JR. Feasibility and validation of real-time patient evaluations of internal medicine interns' communication and professionalism skills. Journal of graduate medical education. 2014;6(1):71-7.
20. She M, Li Z, Rau P-LP, editors. Physician Communication Behaviors that Predict Patient Trust in Outpatient Departments. International Conference on Cross-Cultural Design; 2015: Springer.
