# Supplementary material for: Eliciting the Impact of Digital Consulting for Young People Living With Long-Term Conditions (LYNC Study): Cognitive Interviews to Assess the Face and Content Validity of Two Patient-Reported Outcome Measures
Source: J Med Internet Res. 2018 Oct 11;20(10):e268. doi: 10.2196/jmir.9786 (PMC6231804; doi:10.2196/jmir.9786)
Supplement: Multimedia Appendix 2 [file jmir_v20i10e268_app2.pdf]

Multimedia Appendix 2. Clinician and young people's face and content validity assessments of the patient activation measure 13 and 22 items.

Item number	Item	Number of patients (P) and clinicians (C) in agreement with relevance of item for evaluating digital consulting; P (n=2 or 3 per item); C (n=6 per item)	Number of patients (P) and clinicians (C) able to appraise the question with ease ; P (n=2 or 3 per item); C (n=6 per item)	Illustrative quotes [condition, clinic code, participant role, and participant code]
[a]1	When all is said and done, I am the person who is responsible for managing my health condition	P3; C5	P3; C2	*The patient would be in charge of it and these [emails/texts] are tools which would help you with that*. [Diabetes 2, consultant 01]
2	Taking an active role in my own health care is the most important factor in determining my health and ability to function	P2; C6	P2; C5	*Well one of the problems is that sometimes when I do see patients is they will say, ah I wish I had more frequent appointments so that I can get a kick in my backside to basically get more engaged with the whole process. Because sometimes they are so engrossed with other day-to-day stuff actually, so having digital communication in between is an added bonus compared to face-to-face*. [Diabetes 1, consultant 04]
3	I am confident that I can take actions that will help prevent or minimize some	P2; C6	P2; C5	*I would say agree, because they might then use technology just to, kind of, check in with things like that.*

	symptoms or problems associated with my health condition			*So, for example, sending a message or an email to say, I've got this, what should I do. And then they might get a response back, so I agree with that one*. [Cancer 1, young people's worker 04]
4	I know what each of my prescribed medication does	P2; C4	P2; C4	*Well I did text [name] recently actually, my nurse to ask about one [medication] because I just... they told me that they were reviewing it and they never gave me it so I wasn't sure if they'd just been missed out or something, but I spoke to her and then she was fine with it, she just told me that it was something I didn't need anymore.* [Cancer 2, young person 10]
5	I am confident that I can tell when I need to get medical care and when I can handle the health problem myself	P2; C5	P1; C4	*I often get text messages from them saying, I don't feel very well, have you got space in your clinic. Or they'll text and they'll say, when they've been showering and things, they've found lumps and bumps....so they've been able to get medical care we get them straight into the next available clinic.* [Cancer 1, advanced nurse practitioner 02]
6	I am confident I can tell my health care provider concerns I have, even when he or she does not ask	P3; C6	P2; C4	*I've come to them if I've had things that I haven't been too sure about, or if I'm panicking about something. So I'll email them or text them. Or if something's*

				going wrong and I don't know what to do about it, or if I'm panicking, I'll go and I'll email them, and I'll ask for their advice. [Inflammatory bowel disease 2, young person 19]
7	I am confident that I can follow through on medical treatments I need to do at home	P3; C4	P2; C5	*It's just keeping up with my tablets. So it's up to me. Again, if I needed them or if I had any concerns, I'd just email them, and it would get through straight away*. [Inflammatory bowel disease 2, young person 19]
8	I understand the nature and causes of my health condition/s	P2; C5	P1; C4	*Yeah. I mean it's [digital communication] reinforcing, that's what I would say, more sort of you know, how they are getting on with it, how the progress is. So in chronic disease the important thing is to keep them on track, and you do need to have a constant sort of a, I would say nudge to keep them into that sort of way, and where is that individual going with their health condition with regard to complications and all that sort of thing*. [Diabetes 1, consultant 04]
9	I know the different medical treatment options available for my health condition	P1; C4	P1; C5	*I would agree on that. Again I think that's the bit which most certainly can be done, I would have thought with the help of technology, definitely*. [Diabetes 2, consultant 01]
10	I have been able to maintain the lifestyle	P2; C5	P1; C5	*In the four years I've taken on changes, I've done*

	changes for my health that I have made			*certain things differently, to keep myself healthy. So they'll say, giving certain bits of sport a miss, or giving certain bits a miss won't be too bad in the long run. So keep yourself safe and healthy. Well that's when I'd probably use my phone. Normally it's an email, and then I'll probably text as well.* [Inflammatory bowel disease 2, young person 19]
11	I know how to prevent further problems with my health condition	P1; C4	P2; C4	*I was just thinking again, with young people, that's a big thing that we're trying to do. To kind of, before things go too wrong they contact us, and then we try to pre-empt future difficulties.* [Liver psychologist 01]
12	I am confident I can figure out solutions when new situations or problems arise with my health condition	P2; C5	P1; C5	*In a way that myself, if I wasn't sure about something, I probably wouldn't know what to do, depending on what it was. So I think this is where it comes in where, obviously the text contact with [name] that I have on the ward, and my phone access to the ward. I think that would be key in finding a solution for it or, you know, sorting it out from there*. [Cancer 2, young person 10]
13	I am confident that I can maintain lifestyle changes, like diet and exercise even during times of stress	P2; C4	P1; C5	*Again, it's going back to that would we reasonably think that digital communication can actually affect that. Yeah, I think so actually.*

				Strongly agree, and I'm thinking that often, it's at times of stress that people contact us. Like the two in the morning, I'm at university and I don't know what to do. [Liver psychologist 01]
				If I was stressed I know that they have psychologists and things here, and I could get in touch with one of those. [Cystic fibrosis, young person 10]
[b]14	I am able to handle problems of my health condition on my own at home	P2; C4	P1; C3	*Well it depends on what it was, obviously. I think I'd probably agree with that, to the point that I think you can deal with minor things on your own at home. But as soon as your temperature spikes, or as soon as you realise that you might be coming down with something, then I think it's referring to the ward or your clinical nurse, which is key there. Yeah, so I'd do that by text*. [Cancer 2, young person 11]
15	I am confident I can keep my health condition from interfering with things I want to do	P2; C6	P2; C4	*I'd say in the major part yes, I think I can. Mainly because it's quite mild for me and it doesn't tend to get bad. And when it does I'm kind of experienced and I know what to do, and I know to contact them [via email first], if it's bad, to get the right prescription.* [Inflammatory bowel disease 2, young person 07]

16	Making the lifestyle changes that are recommended for my health condition are too hard to do on a daily basis	P1; C4	P1; C5	*I think sometimes when they've been through all the treatment plans that they've got, and then they finish and then they've got to start focusing on getting back into the real world, engaging with colleges and universities, starting back with their friends. So again, it's through, sort of, Facebook where they might be able to ask others that have been through it what, you know, lifestyle changes that they've been through and how did they get on. And again, if they've got any worries or concerns, they can text and they can come in and see us as soon as possible.* [Cancer 1, advanced nurse practitioner 02]
17	I am confident that I can find trustworthy sources of information about my health condition and my health choices	P2; C5	P2; C5	*Well if there's any specific questions I can ask at my appointment, but that is only every three months. So it might be useful for the little niggling things in between to be able to like, text someone or message someone more easily. And less formal. Yeah*. [Inflammatory bowel disease 2, young person 07]
18	I am confident that I can follow through on recommendations my health provider makes such as changing my diet or taking regular exercise	P2; C5	P1; C4	*I would say yes, that's agree with that one as well. Because again, things like it, may be that the health provider has talked about perhaps using an app, or actually using technology to,*

				you know, monitor steps or that sort of thing. So they might, the health provider might have even recommended things they can use with technology themselves to, yeah, about diet and exercise in particular because there's loads of stuff out there for that. [Cancer 1, young people's worker 04]
19	I know the lifestyle changes like diet and exercise that are recommended for my health condition	P 2; C5	P 1; C4	*I don't think that [digital clinical communication] would change that much. Because I think, the impression that I've got, is that that's because there's not that much known about how the diet affects it.* [Inflammatory bowel disease 2, young person 07]
20	I know about the self-treatments for my health condition	P1; C3	P0; C4	*Yeah, I'd say agree for that one, because again it might be something that they check via email. They might have had some information face to face and then email to perhaps follow that up.* [Cancer 1, young people's worker 04]
21	I have made the changes in my lifestyle like diet and exercise that are recommended for my health condition	P2; C4	P2; C3	*Because it's about maintenance of the changes isn't it, and hopefully people would be checking in using digital stuff.* [Liver psychologist 01]
22	I am able to handle symptoms of my health condition on my own at home	P2; C3	P2; C4	*Yeah, I think I might email the nurse first, because sometimes they're a bit reluctant to give out steroids,*

				so I'd want to know whether the state I was in was worth me coming in and getting them, I think. [Inflammatory bowel disease 2, young person 07]

[a]Items 1 to 13 are from PAM 13.

[b]Items 14 to 21 are present only in PAM 22 full scale.
