# Supplementary material for: Eliciting the Impact of Digital Consulting for Young People Living With Long-Term Conditions (LYNC Study): Cognitive Interviews to Assess the Face and Content Validity of Two Patient-Reported Outcome Measures
Source: J Med Internet Res. 2018 Oct 11;20(10):e268. doi: 10.2196/jmir.9786 (PMC6231804; doi:10.2196/jmir.9786)
Supplement: Multimedia Appendix 3 [file jmir_v20i10e268_app3.pdf]

Multimedia Appendix 3. Clinicians and young people's face and content validity assessments of the physician humanistic behavior questionnaire.

Item number	Item	Number of patients (P) and clinicians (C) in agreement with relevance of item for evaluating digital consulting; P (n=2 or 3 per item); C (n=6 per item)	Number of patients (P) and clinicians (C) able to appraise the question with ease; P (n=2 or 3 per item); C (n=6 per item)	Illustrative quotes [condition, clinic code, participant role, participant code]
1	Follows through on problems	P3; C6	P3; C5	*Because obviously the email is the first point of contact in every single time that I've had a relapse, or anything like that. It's always been email I've contact them by*. [Inflammatory bowel disease 2, young person 21]
2	Is truthful and honest with me without avoiding the issues	P2; C5	P1; C5	*I think it's one of the reasons people get nervous about it, because you can't flower. You can be very careful about the language you use, but ultimately it is in black and white, written down there*. [Liver psychologist 01]
3	Is in a hurry	P0; C5	P2; C4	*It's like if I've ever texted my nurse or [name] or I've ever rang them, you usually get a reply in the next hour depending on if they're free, and if you ring them they almost always pick up straight away unless they're in a meeting or something.* [Cancer 2, young person 10]

4	Expresses concern for my feelings and needs, not just my physical status	P3; C6	P2; C4	*I think again it’s just kind of the fact of thing that you do respond to that in a way means actually that you are concerned for their feelings and their needs, because otherwise we wouldn't be using it. Otherwise we would say, come to the clinic next week and we'll talk about it then. So I think the fact that you provide that type of service, I would think it tells you that you're kind of concerned.* [Liver consultant 05]
5	Comforts or reassures me and my family	P2; C6	P1; C5	*Because I think that often the technology is used outside of say a clinical setting and I think it's important for the young person or family to be able to say, does that comfort or reassure them, or actually do they perhaps find it a bit intrusive. So it gives their perspective on how the technology makes them feel.* [Cancer 1, young people’s worker 04]
6	Asks how I am doing	P2; C5	P2; C4	*They ask me, how are you doing, all the time. Like I say [name] and [name] have rung me since I've started my treatment just to make sure I'm getting on okay.* [Cancer 2, young person 10]
7	Keeps his or her promises to me	P0; C4	P1; C5	*Well I mean I think I'm neutral on that because you know, you don't know what the expectations are. So you know, it depends for what the promise was made, whether it's realistic or not, in what setting the promise was made. So I would*

				say it's a neutral thing as to what...I mean it depends on the goal setting and the promises doesn't it, so I would be neutral. [Diabetes 02, consultant 1]
8	Pays attention to concerns or requests that I feel are important	P2; C6	P1; C5	*And I'm sure if I was texting [name] about something, then she'd find out what it was. You know, if she was at work, or a couple of hours later, and text me back with a solution, or just saying that she's registered that and she's going to do something about it*. [Cancer 2, young person 11]
9	Explains and clarifies information for me	P2; C5	P2; C5	*Again that's the strength of digital technology where you can do a lot of that, so well you pick up the phone maybe or use Skype to do that, so in a diabetes setting that's the right thing*. [Diabetes 2, consultant 01]
10	Answers my questions	P3; C5	P3; C4	*Strongly agree, answers my questions. Both physically and digitally. Every time I go into clinic, if I do have a question, I can always knock on [name]'s door and ask her something. Or I can just email her. Even while I'm there I can email her, and she may even reply before I leave.* [Inflammatory bowel disease 2, young person 21]
11	Makes uncaring remarks or does things I find offensive	P0; C4	P2; C5	*I would say agree with that one. I think that it's important to include so that people have got a platform to voice that if they feel that that's how that communication makes them feel. But I guess as well that the answer to that can be a bit*

				subjective can't it, so... and I think it's to do with the intention. But equally I think intention can get miscommunicated via email and text. Yeah, so think that's important. [Cancer 1, specialist nurse]
12	Discussed the options for my treatment	P1; C3	P2; C3	*I don't know that you should be getting into those kinds of conversations over digital communication. I think there are some things that have to happen, either face-to-face or at least over the phone.* [Liver psychologist 01]
13	Uses terms that I can understand	P3; C5	P1; C3	*I'd say it's more difficult to understand in clinic than it is to understand online. Especially when you're talking to consultants, you know, they are professionals. They do like to get into the nitty gritty science of stuff. And [name] doesn't.* [Inflammatory bowel disease 2, young person 21]
14	Includes me in decisions and choices about my care	P2; C6	P1; C4	*You know for things like this, he rang me and text me today and asked me if it was okay to give you my number. And I think, you know, they included me in making that decision, they didn't just give you the number. As far as including me in other things, like he always invites me to social events instead of saying that I'll come along, etc., which I know he wouldn't do.* [Cancer 2, young person 11]
15	Arranges for adequate privacy when examining	P2; C3	P2; C5	*Even with the phone calls and stuff like they always ask I'm okay to speak or if I want to go*

	or talking with me			*somewhere, I can give them a ring back or whatever if I ever wanted to go somewhere private like when they rang me up about going back to hospital again. It was nice to know that like they didn't want to tell me bad news in front of everybody*. [Cancer 2, young person 10]
16	Has a neat, clean, well-groomed appearance	P3; C1	P0; C3	*Yeah, because I think if you're using Skype, I wouldn't Skype someone at home sitting in my pyjamas if I was working from home for the day.* [Cancer 1, young people's worker 04]
17	Is short tempered or abrupt with me and my family	P0; C3	P0; C4	*I think sometimes people can be sensitive in terms of like if you type stuff all in caps, some people think you are shouting at them or being rude, and aggressive. So I think yeah, being able to come across as short tempered or abrupt you can get in the tone of something that's written.* [Cancer 1, young people's worker 04]
18	Makes changes in my treatment without telling me	P0; C3	P0; C3	*I think it's [digital clinical communication] largely used within our service about communicating changes in treatment. So it's an effective way of doing that.* [Liver psychologist 01]
19	Doesn't rush or spend too little time with me	P3; C2	P2; C4	*Because they can sort of get back to me when, rather than say, well I don't know now. They can say, well I don't know the answer, but I can put you in touch with someone who does, or get back to you.* [Cystic fibrosis, young person 10]
20	Asks if I need	P2; C5	P1; C6	*I think I've actually had, by text*

	anything or what he or she can do for me			*actually, me and [name] were having a back and forth about writing a letter to my university. So, you know, at that point in time she was asking if she needed to do anything, and I said yeah, it would be nice if you could send a letter. And she did that*. [Cancer 2, young person 11]
21	Asks how I want to be addressed and then greets me in that way	P1; C3	P1; C4	*I've always been called by my nickname or just [name] always manages to come up with random nicknames that are playful, but you know, you deal with it [laughs]. Even in text they always address me as [name] instead of [name] and stuff like that if I've asked them to.* [Cancer 2, young person 10]
22	Seems knowledgeable and concerned about me and my case	P3; C5	P2; C4	*I think I had loads of voice mails saying, you know, there's a bed ready, are you okay, are you still happy to come in, are you well. And they kept getting in touch until I finally got hold of them and said, look, I can't come in at the minute, and they were happy for that. But until I got in touch, they kept on until I got in touch. So they cared.* [Cystic fibrosis, young person 10]
23	Asks questions about my symptoms	P2; C6	P2; C4	*Well, [name] obviously asks me if I've had any more symptoms and things, if I'm coming down with a cold when we speak about...obviously I went to [place] the other week actually, and she text. Well I let her know afterwards, she text me and said, you know, did you have*

				any symptoms or have you come back with a cold, or anything like that? And that was all by text. [Cancer 2, young person 11]
24	Treats me with too intimate or personal a manner	P0; C2	P2; C3	*People might think the opposite because I've got patients who, even though I still put, you know, write it like a letter so put, Dear so and so, you know, I just sign off with best wishes and my name, I've still got patients who would be really informal with me, and will put multiple kisses at the end, and I never reciprocate, but I don't address the fact that they're doing that either. So they might just think I'm horribly cold because I don't, I don't know. The other way round would be really shocking wouldn't it, if you were putting lots of kisses towards a patient.* [Liver psychologist 01]
25	Asks me how I feel about my problems	P3; C4	P2; C4	*Because often that's why the technology might be being used.* [Cancer 1, young people's worker 04]
